# Supplementary material for: A Biopsychosocial Model of Sex Differences in Children’s Eating Behaviors
Source: Nutrients. 2019 Mar 22;11(3):682. doi: 10.3390/nu11030682 (PMC6470823; doi:10.3390/nu11030682)
Supplement: Supplementary file 1 [file nutrients-11-00682-s001.pdf]

Supplementary Materials

I. Evidence of sex differences on eating behavior in children

I.e. Sex differences in child appetitive traits and Eating in the Absence of Hunger:

Table S1. Parent Report for Child Eating Behaviors Questionnaire

	Males	Females		
	Mean (SD)	Mean (SD)	p^A	d^B
Slowness in Eating	2.79 (0.66)	2.83 (0.80)	0.634	0.06
Satiety Responsiveness	2.90 (0.56)	2.87 (0.65)	0.672	0.05
Emotional Under Eating	2.73 (0.74)	2.69 (0.70)	0.681	0.05
Food Fussiness	2.96 (0.81)	2.79 (0.87)	0.103	0.20
Enjoyment of Food	3.70 (0.65)	3.83 (0.65)	0.106	0.20
Food Responsiveness	2.54 (0.63)	2.66 (0.78)	0.172	0.17
Desire to Drink	2.73 (0.86)	2.60 (0.93)	0.233	0.15
Emotional Overeating	1.87 (0.62)	2.02 (0.72)	0.073	0.22

A: p-values reflect two-samples t-tests, uncorrected for multiple comparisons
B: Cohen's d

II. Biopsychosocial contributions to sex differences

II.a. Neural differences in the response to food cues

Methods:

Image Acquisition Parameters

Participants were imaged with a Siemens MAGNETOM Trio 3T MRI scanner and 12-channel head coil. Structural scans were acquired over 4:34 minutes using a T1-weighted magnetization-prepared rapid gradient-echo sequence (TR = 1650ms, TE = 2.03ms, flip angle = 9°; 160 sagittal slices with a 1.0 mm thickness; 1x1x1mm voxels, FOV=256x256mm). Functional scans were acquired using a T_2*-weighted echo planar-imaging (EPI) sequence (TR = 2000ms, TE = 25ms, flip angle = 90°, FOV= 220x220mm, 3x3x3mm voxels, 33 interleaved descending 3mm slices). EPI acquisition was aligned with AC-PC and adjusted vertically to optimize signal in temporal and cerebellum. Head movement was minimized with padding between the head and coil. In-scan prospective movement correction (PACE) was used to assist with the motion-induced effects during acquisition by adjusting the slice positioning.

Preprocessing Steps

Preprocessing included non-linear warping of each participant's anatomical scan into standard space using AFNI's MNI152_2009_template_SSW template. Using the afni_proc.py python script, functional images were despiked, corrected for slice timing, aligned to the EPI volume with the minimum outlier fraction, co-registered with each subject's anatomical scan, and warped into standard space. Images were smoothed with a Gaussian filter set at 6 mm full-width at half maximum.

For each participant, a General Linear Model included 6 parameters of interest and 12 parameters of no interest. The 6 parameters of interest were modeled using block duration and onset for the 6 image conditions: (1) high ED-large portion, (2) high ED-small portion, (3) how ED-Large portion, (4) how ED-small portion, (5) furniture, (6) scrambled images. The 12 regressors of no interest included 6 head motion parameters and their derivatives. The following first-level contrasts were generated for each subject: (1) high-ED, large portion > low-ED, large portion, (2) high-ED, large portion > low-ED, small portion, and (3) high-ED (large and small portion) > low-ED (large and small portion).

Scan volumes were censored if (1) adjacent functional volume had a Euclidean norm of the motion derivative that exceeded 1 mm or (2) more than 10% of voxels in the masked brain were outliers. A run was excluded from analyses if it had >10% of volumes censored for motion per run or >20% of volumes censored for motion in any single food block for a run. Participants were excluded from analyses if they had fewer than 4 acceptable functional runs.

Since whole brain coverage was not achieved during acquisition of functional images, group analyses were constrained to voxels where at least 80% of participants had coverage.

Analyses:

Portion Size: Using the ED contrasts for each portion size, a repeated-measures analysis of variance using 3dMVM [1] examining a portion size X sex interaction revealed no evidence for a main effect of sex, main effect of portion size, or a portion size X sex interaction. Therefore, the remaining group analyses focused on the ED contrast collapsed across portion size. Using a 2-sample *t*-test we found no evidence for a sex difference in neural response to high- compared to low-ED foods. We next conducted a sex X BMI z-score analysis of covariance (ANCOVA; [1]) to examine if neural responses to high-ED compared to low-ED foods show a similar sex X adiposity interaction.

BMIz X Sex: In addition to the BMIz X child sex ANCOVA with the overall ED contrast (high ED – low ED), three additional group models were run to confirm results. First, to check if the results were due to the first-level overall ED contrast, a Sex X BMIz linear mixed effects model that included the ED contrasts for each portion size and controlled for random effects of participant was conducted using 3dLME.

Second, we tested to see if results were independent of pre-and post-MRI perceived fullness ratings. Before and after the MRI, children rated their perceived fullness levels using a 150-mm visual analog scale, which depicted a stick figure with a rectangular stomach[2]. Males and females significantly differed in their pre-MRI (Male: Mean = 26.53, SD = 30.60; Female: Mean = 50.79, SD = 39.89; $t(41)=2.19$, $P=0.034$) and post-MRI (Male: Mean = 22.95, SD = 24.22; Female: Mean = 45.42, SD = 39.92; $t(39)=2.28$, $P=0.028$) fullness ratings. Pre- and post-fullness ratings were independently tested as covariates in two separate BMIz X sex multivariate models.

Multiple comparisons were controlled at $p<0.05$ using Monte-Carlo simulations through AFNI's 3dFWHMx and 3dClustSim [3] using the mixed autocorrelation function to better model non-Gaussian noise structure (NN=2, 2-sided, $P < .001$, k=29). See Figure S1 for overlap of significant clusters across analyses.

Results:

Similar to the Sex X BMIz multivariate model with the overall ED contrast, none of the additional models revealed main effects of child sex or BMIz on neural responses to high-ED compared to low-ED food cues. All three models did, however, show significant BMIz x Sex interactions in a pattern consistent with the results reported in the main text (see Figure S1 for overlap of significant BMIz x Sex clusters across all models):

1. Mixed linear effects model, with ED contrasts (high ED – low ED) for each portion size: right fusiform gyrus($F(1,39) = 20.29$ peak: $x = 28.5$, $y = -52.5$, $z = -10.5$, $k = 61$).

2. BMI-z X Sex ANOVA on overall ED contrast, with pre-scan fullness covariate: right superior tempral gyrus ($F(1,38) = 25.47$ peak: $x = 37.5$, $y = -37.5$, $z = 7.5$, $k = 172$).

3. BMI-z X Sex ANOVA on overall ED contrast, with post-scan fullness covariate: right fusiform gyrus ($F(1,38) = 21.00$ peak: $x = 28.5$, $y = -52.5$, $z = -10.5$, $k = 81$;) and right superior temporal gyrus ($F(1,38) = 23.66$ peak: $x = 37.5$, $y = -37.5$, $z = 7.5$, $k = 47$). These two distinct clusters overlap with the cluster revealing a BMIz x Sex interaction when fullness covariates were not included in the model.

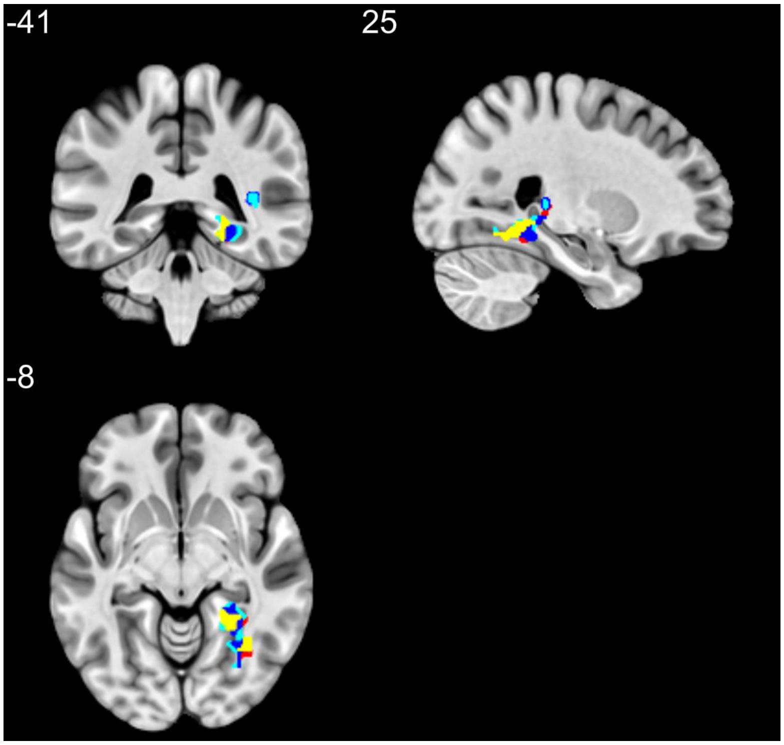

Figure S1. Overlap of significant clusters from the four analyses: (1) ANCOVA with overall ED contrast [red], (2) Linear mixed effects model with ED contrasts for each portion size [yellow], (3) ANCOVA with overall ED contrast and pre-MRI fullness covariate [cyan], (4) ANCOVA with overall ED contrast and post-MRI fullness covariate [blue].

1. Chen, G.; Adleman, N. E.; Saad, Z. S.; Leibenluft, E.; Cox, R. W., Applications of multivariate modeling to neuroimaging group analysis: a comprehensive alternative to univariate general linear model. *NeuroImage* **2014,** *99*, 571-588.
2. Keller, K. L.; Assur, S. A.; Torres, M.; Lofink, H. E.; Thornton, J. C.; Faith, M. S.; Kissileff, H. R., Potential of an analog scaling device for measuring fullness in children: Development and preliminary testing. *Appetite* **2006,** *47* (2), 233-243.
3. Cox, R. W., AFNI: software for analysis and visualization of functional magnetic resonance neuroimages. *Comput Biomed Res* **1996,** *29*, 162-73.
